# Usefulness of aqueous and vitreous humor analysis in infectious uveitis

**DOI:** 10.6061/clinics/2020/e1498

**Published:** 2020-01-20

**Authors:** Helen Nazareth Veloso dos Santos, Eduardo Ferracioli-Oda, Thaisa Silveira Barbosa, Camila Sayuri Vicentini Otani, Tatiana Tanaka, Luciane de Carvalho Sarahyba da Silva, Guilherme de Oliveira Lopes, Andre Doi, Carlos Eduardo Hirata, Joyce Hisae Yamamoto

**Affiliations:** I Servico de Uveites, Departmento de Oftalmologia, Hospital das Clinicas HCFMUSP, Faculdade de Medicina, Universidade de Sao Paulo, Sao Paulo, SP, BR; II Secao de Biologia Molecular, Hospital das Clinicas HCFMUSP, Faculdade de Medicina, Universidade de Sao Paulo, Sao Paulo, SP, BR; III Secao de Citometria de Fluxo, Divisao do Laboratorio Central DLC, Hospital das Clinicas HCFMUSP, Faculdade de Medicina, Universidade de Sao Paulo, Sao Paulo, SP, BR

**Keywords:** Polymerase Chain Reaction, Infectious Disease, Uveitis, Aqueous Humor, Vitreous Humor

## Abstract

**OBJECTIVE:**

To evaluate the role of intraocular fluid analysis as a diagnostic aid for uveitis.

**METHODS:**

Twenty-eight samples (27 patients including 3 HIV-infected patients) with active (n=24) or non-active (n=4) uveitis were submitted to aqueous (AH; n=12) or vitreous humor (VH) analysis (n=16). All samples were analyzed by quantitative PCR for herpes simplex virus (HSV), varicella zoster virus (VZV), cytomegalovirus (CMV), Epstein-Barr virus (EBV) and Toxoplasma gondii.

**RESULTS:**

The positivity of the PCR in AH was 41.7% (5/12), with 50% (2/4) in immunocompetent and 67% (2/3) in HIV+ patients. The positivity of the PCR in VH was 31.2% (5/16), with 13% (1/8) in immunocompetent and 50% (4/8) in immunosuppressed HIV negative patients. The analysis was a determinant in the diagnostic definition in 58% of HA and 50% of VH.

**CONCLUSION:**

Even in posterior uveitis, initial AH analysis may be helpful. A careful formulation of possible clinical diagnosis seems to increase the chance of intraocular sample analysis being meaningful.

## INTRODUCTION

Uveitis can cause significant visual loss, accounting for up to 20% of the cases of legal blindness (1,2) and can be related to an infectious or noninfectious cause, the distribution of which varies according to the country and region studied (3,4). In Brazil, infectious uveitis accounts for 46% to 63% of uveitis in tertiary services (5); similarly this high rate is observed in Argentina (48%), in India (55%) and in the United States (26 to 36%), whereas in some countries infectious uveitis is less frequent, e.g., in Japan (13 to 19%) and in China (5%) (6-8). The most frequent microorganisms related to infectious uveitis are *Toxoplasma gondii*, *Treponema pallidum*, *Mycobacterium tuberculosis* and herpes virus family ((*herpes simplex* virus (HSV), *herpes zoster* virus (VZV), cytomegalovirus (CMV), *Epstein-Barr* virus (EBV)) (9,10). Noninfectious uveitis, on the other hand, comprises a group of heterogenous disorders that may be either confined to the eye or associated with systemic symptoms (11).

Techniques based on polymerase chain reaction (PCR) and biomarkers have been studied and applied in intraocular fluids since the end of the 20^th^ century (12,13), with the aqueous humor analysis in cases of posterior uveitis described as useful for the etiological diagnosis in up to 29% of the cases studied, according to Rothova et al. (14). However, in Brazil, as well as in many countries, PCR of ocular fluid is not included in a daily clinical practice among uveitis specialists, with few related studies (5,12). Thus, we report our experience in the use of PCR technology in the analysis of aqueous and vitreous humor in patients with uveitis, emphasizing the importance of a detailed elaboration of the clinical hypothesis to the success of a final diagnosis.

## METHODS

This prospective study included 27 patients (28 samples), including 3 HIV-infected patients who presented in the Uveitis Service, Hospital das Clinicas HCFMUSP, Faculdade de Medicina, Universidade de Sao Paulo, Sao Paulo, SP, BR, between March 2014 and October 2017, with active (n=24) or non-active (n=4) uveitis and submitted to analysis of aqueous humor (AH; n=12) or vitreous humor (VH; n=16). Intraocular fluid analysis was indicated in selective cases, e.g. atypical cases and/or evolution, intraocular tumor suspicion, and severe inflammation with imminent risk of visual loss and immunosuppression. The average time between clinical onset and aqueous/vitreous tap was 27 months (range: 1.5-72 months). Controls consisted of AH samples from patients undergoing cataract surgery (n=10) and of VH samples from patients undergoing vitrectomy (n=4); control cases for vitreous samples had disorders not related to infectious or inflammatory causes, i.e., epiretinal membrane and retinal detachment. Patients with diabetes and age-related macular disease were also excluded from the control group. This study was approved by the Institutional Ethics Committee. It adheres to the tenets of the Declaration of Helsinki.

All patients underwent complete ocular examination including measurement of the best corrected visual acuity, anterior and posterior segment biomicroscopy, applanation tonometry, and indirect binocular ophthalmoscopy. Complementary exams such as retinography, fluoresceinangiography (FA), indocyanine green angiography (ICGA), spectral-domain optical coherence tomography (OCT), ocular ultrasound, electroretinogram and visual field tests were performed whenever indicated for better characterization and follow-up of patients. Based on the initial clinical diagnosis, laboratory and systemic investigation was carried out for infectious or noninfectious diagnosis.

Active disease was defined as the presence of 1+ or more cells in the anterior chamber and cells in the anterior vitreous, 1+ or more vitreous haze, exudative chorioretinitis or retinochoroiditis lesions, optic neuritis, retinal vasculitis and macular edema. Posterior segment abnormalities were better characterized by FA and OCT.

For anterior chamber paracentesis, uveitis should be active with a minimum of 2+ cells. Diagnostic vitrectomy was performed when indicated. Vitrectomy had diagnostic (n=4), diagnostic and therapeutic (n=9) and therapeutic (n=3) indication.

All samples were analyzed by quantitative PCR for *Toxoplasma gondii*, HSV, VZV, CMV and EBV. DNA was extracted from samples using QiAamp^®^ DNA mini kit (Qiagen Biotecnologia Brasil) on the Qiacube platform. From March 2014 to May 2017, real time PCR for these agents, except for VZV was carried out according to previous studies (12,15,16) and validated in our institution. PCR for VZV was qualitative PCR.(16) From May to October 2017, a multiplex PCR test, FTD Neuro9 (Fast track diagnostics, Siemens Healthineers Company), an in vitro diagnostic test certified by European Community and validated by the College of American Pathologists was used, optimizing the small sample amount. The multiplex PCR test includes HSV, VZV, CMV and EBV (15-17). For *Toxoplasma gondii*, real time PCR using SYBR green DNA-dye-complex was implemented (18).

Vitreous samples complementary analysis was defined according to the suspected diagnosis.

Main outcomes consisted of PCR positivity and whether the PCR results changed the clinical management of each case. PCR results were considered determinant when the results were accordingly or changed the initial clinical diagnosis.

A descriptive analysis was included. Fisher’s exact test was used for relation analysis between PCR positivity and immune status; significance <0.05.

## RESULTS

The positivity of the PCR in AH was 41.7% (5/12), 50% (2/4) in immunocompetent and 37.5% (3/8) in immunosuppressed patients (*p*>0.999, Fisher’s exact test); among immunosuppressed, 67% (2/3) were HIV+patients. The clinical diagnosis of those with AH PCR positive samples included the following: for CMV, a 19-year-old male with recurrent unilateral anterior hypertensive uveitis with clinical characteristics of Posner-Schlossman syndrome; for HSV, a 48-year-old female with acute retinal necrosis (ARN); for *T.gondii,* 2 HIV-positive patients, a 36-year-old male with ARN and a 70-year-old male with diffuse uveitis; and, for EBV, an eight-year-old girl with poli articular juvenile idiopathic arthritis and chronic anterior uveitis refractory to prednisone, mycophenolate mofetil and infliximab infusion.

The positivity of the PCR in VH was 31.2% (5/16), which was 13% (1/8) immunocompetent and 50% (4/8) in immunosuppressed HIV-negative patients (*p*=0.282, Fisher’s exact test). *T.gondii* were detected in 4 samples: in a 66-year-old male with lymphoma with recurrent urinary tract infection and diffuse uveitis; in a 56-year-old woman with eosinophilic vasculitis in use of high dose of oral prednisone and posterior uveitis; in a 64-year-old woman with T-cell lymphoma and posterior atypical uveitis; and in a 64-year-old woman with granulomatous diffuse uveitis. CMV was detected in a 65-year-old male with lymphoma and post bone marrow transplantation with diffuse uveitis. Cytology and flow cytometry were performed in seven vitreous humor samples, being negative for atypia or for monoclonal expansion of T or B cells. Culture was positive for *C. albicans* in two cases: a 66-year-old male with lymphoma who was PCR positive as described previously and a 71-year-old woman with central venous access for recurrent pulmonary infection and chronic obstructive pulmonary disease with fungal endogenous endophthalmitis (Table 1).

**Figure 1:**
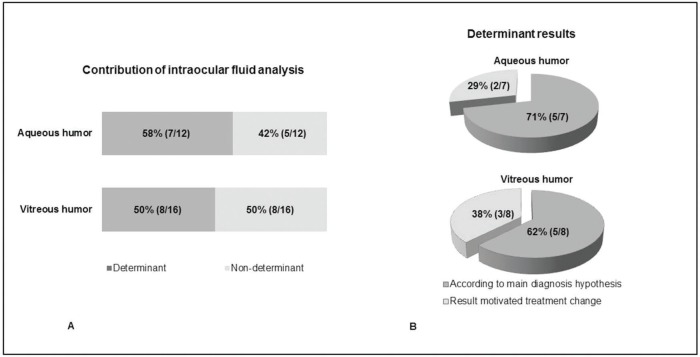
A. Contribution of intraocular fluid analysis in patients with uveitis; B. Determinant results found for aqueous and vitreous humor. The results were considered determinant when they were in accordance with the initial clinical hypothesis (B. dark gray) or changed the initial clinical diagnosis (B. light gray).

The analysis of the intraocular fluid was determinant in the diagnostic definition in seven out of twelve samples of AH (58%) and eight out of 16 samples of VH (50%) (Figure 1). Concerning the determinant cases, a previous clinical diagnosis was confirmed in 71% of cases and in 62% of cases, AH and VH samples, respectively; and a different diagnosis was made in 29% and 38% of AH and VH samples, respectively.

A summary of similar studies in the literature, including the present study results, with AH and/or VH analysis in patients with uveitis is represented in Table 2. No statistically significant difference between PCR positivity and immune status was found and no positivity was observed in any control samples.

## DISCUSSION

Our study aimed to evaluate the role of intraocular fluid analysis as a diagnostic aid for uveitis. Intraocular fluid analysis was indicated in selective cases, e.g., atypical cases and/or evolution, intraocular tumor suspicion, severe inflammation with imminent risk of visual loss and immunosuppression. We verified that it was a determinant in the diagnostic definition of 58% AH and in 50% of VH; our positivity for PCR in AH and VH was 41.7% and 31.2%, respectively.

Several studies pointed out the value of vitreous sample analysis for diagnostic purposes in case of posterior/diffuse uveitis (12,19); nevertheless, Rothova et al. (14,20) have shown that an initial AH analysis can be useful and even avoid the need for a more invasive procedure, such as vitrectomy *pars* plana. These authors verified that 15 out of 44 patients (34%) with posterior uveitis had their etiological diagnosis confirmed by AH analysis. In a similar study carried out in Thailand, the same authors observed PCR positivity for AH and VH of 35% and 19%, respectively, in cases of posterior uveitis or panuveitis (21). These data are still in agreement with several studies of other authors (22,23). Our findings also corroborate the usefulness of initial AH analysis in cases of posterior uveitis, i.e. in three out of five patients (60%) with posterior uveitis, AH analysis was determinant for the final diagnosis (two cases of ARN with PCR positive each for HSV and *T. gondii*, and one case of diffuse uveitis with PCR positive for *T. gondii*).

Importantly, uveitis secondary to toxoplasmosis may have atypical presentations and, in this situation, PCR analysis of intraocular fluid is an important diagnostic tool (24). Indeed, in our series of cases, there were two cases with a final diagnosis of ARN caused by *T. gondii*. Talabani et al. evaluated the contribution of immunoblotting, real-time PCR and Goldmann-Witmer coefficient (GWC) to diagnosis of atypical toxoplasmic retinochoroiditis in 54 patients (25). Unlike immunoblotting and the GWC, the PCR results of AH were not influenced by the longer interval between onset and paracentesis. The presence of positive PCR results in the late phases of ocular toxoplasmosis, in turn, could be attributed to intraocular slow release of *T*. *gondii* tachyzoites. The analysis of AH, therefore, in cases of atypical presentation uveitis, such as in ocular toxoplasmosis may be an easy diagnostic tool that can be used for initiating appropriate treatment at a timely stage.

VH analysis, in turn, is useful in atypical cases and in cases in which there is suspicion of malignancy, and it is very helpful diagnosing infections with multiple pathogens (12,26). In our series, we identified a patient whose vitreous sample was PCR positive for *T.gondii* and also culture positive for *Candida albicans:* a 66-year-old male patient with bilateral diffuse uveitis and previous treatment for lymphoma, use of urethral catheter and recidivant urinary tract infections at the time of VH analysis. Scheepers et al. (27) described, in 159 patients, who were 142 HIV-positive, five patients that tested PCR positive for more than one pathogen: four patients were CMV and VZV positive, and one patient was CMV and *T. gondii* positive (23). Multiple infections in patients with some type of immunodeficiency have been previously reported, mainly due to reactivation of latent infection (23,28,29).

In the literature, PCR positivity for AH and for VH from patients with uveitis (including anterior, posterior, intermediate and diffuse uveitis) varied from 19 to 57% and from 19 to 30%, respectively (12,14,21,27,30-35). In our study, the PCR positivity was similar to the best results in the literature, 41.7% for AH and 31.2% for VH (Table 1). Several factors may influence PCR positivity (23). These include: a. prevalence of the disease in the tested population (32); b. pathogen characteristics, e.g., after disease onset, viral DNA can be detected earlier than *T. gondii* DNA (36); c. sample characteristics (AH *versus* VH; volume), adequate conditions for sampling collection and transportation (36); d. PCR assay (e.g., in house, multiplex) (33); e. activity of disease; f. patient’s immune status and (14); g. previous specific or nonspecific treatment, reducing pathogen load (32).

While sampling ocular fluid for diagnostic purpose, disease activity status seems to be relevant and could contribute to the high PCR positivity obtained in our study. Indeed, AH analysis was carried out only if there was at least 2+ cells in the anterior chamber (37); while 12 of 16 patients with VH analysis did have active uveitis at the time of analysis. Among the four VH samples from patients with inactive uveitis, two had the clinical diagnosis of ARN and negative PCR analysis; in these cases, vitrectomy was indicated to address ocular complications, mainly retinal detachment. High PCR positivity, described by Calvo et al. (34), while analyzing AH of 14 patients with ARN, may be due to sampling all patients at initial presentation.

Careful planning and selecting cases with suspicion of an infectious etiology before sampling ocular fluid may also have contributed significantly to our results (38). Several studies have demonstrated the usefulness of intraocular fluid in uveitis as a determinant of a definitive diagnosis in 57.2% to 76.6% of cases (23,27). Scheepers et al. were able to confirm the pretest diagnosis 34.6% and verified a change in diagnosis in 22.6% of the patients who submitted to the analysis of intraocular fluid through PCR (27). The results found are attributed by the authors, among other factors, to the fact that all patients undergoing intraocular fluid analysis had previously been diagnosed with infectious uveitis. Harper et al., in turn, attributed the results mainly to the fact that PCR analysis was performed prior to a careful clinical study of each patient in addition to a detailed ophthalmological examination (23). In this study, the pretesting hypothesis was confirmed in 57.1% and the diagnosis was altered in 19.5% of the patients. In our study, the analysis of the intraocular fluid was a determinant in the diagnostic definition in seven out of 12 samples of AH (58%) and in eight out of 16 samples of VH (50%). Among those cases in which the PCR analysis was determinant for the final diagnosis, in one third it motivated treatment change. This was remarkable for cases with viral or undefined clinical diagnosis that came about due to toxoplasmosis indicating the importance of ocular fluid analysis in atypical ocular toxoplasmosis.

The main limitation of this study is the small number of patients and, despite being a prospective study, paired AH and VH sampling was not possible. Nevertheless, similar studies in Brazil are scarce and this study demonstrates that intraocular fluid PCR analysis is useful for diagnostic purposes attempting to a previous careful planning in cases with infectious etiology suspicion and to disease activity status.

## AUTHOR CONTRIBUTIONS

Santos HNV was responsible for the data curation, formal analysis, investigation, visualization, manuscript original draft, review and editing. Ferracioli-Oda E, Barbosa TS and Otani CSV were responsible for the data curation, project administration, validation, visualization, manuscript writing, review and editing. Tanaka T was responsible for the conceptualization, data curation, formal analysis, project administration, validation, visualization, manuscript writing, review and editing. Silva LCS and Lopes GO were responsible for the data curation, formal analysis, project administration, validation, visualization, manuscript writing, review and editing. Doi A was responsible for the conceptualization, formal analysis, methodology, supervision, validation, visualization, writing manuscript original drafting, review and editing. Hirata CE was responsible for the investigation, supervision, validation, visualization, writing manuscript original drafting, review and editing. Yamamoto JH was responsible for the conceptualization, data curation, formal analysis, investigation, methodology, project administration, supervision, visualization, writing manuscript original drafting, review and editing.

## Figures and Tables

**Table 1:** Clinical data and polymerase chain reaction (PCR) results of 28 samples of patients with uveitis included in the study.

Description	Aqueous humor	Vitreous humor
N	12	16
**Clinical diagnosis**		
Anterior uveitis, n (%)	4 (33.3)	-
Uveitis associated with JIA!	2	-
Posner-Schlossman syndrome	1	-
Herpetic uveitis	1	
Posterior uveitis, n (%)	5 (41.7)	10 (62.5)
Ocular toxoplasmosis	1	6
Acute retinal necrosis	2	3
Cytomegalovirus retinitis	2	-
Ocular syphilis	-	1
Diffuse uveitis, n (%)	3 (25)	6 (37.5)
Ocular toxoplasmosis	1	1
Intraocular lymphoma	-	2
Fungal endophthalmitis	-	2
Ocular tuberculosis	1	-
Idiopathic	1	1
**PCR positivity**, n (%)	5 (41.7)	5 (31.2)
*T. gondii*	2	4
Cytomegalovirus	1	1
Herpes *simplex* virus	1	-
*Epstein-Barr* virus	1	-
**Clinical impact**, n (%)	7 (58.3)	8 (50)
Changed treatment	2	3
Confirmed clinical diagnosis	5	5
**Immunological status**		
HIV positive	3	0
Others (malignancy, immunosuppressive drug)	1	8
Immunocompetent	8	8

! JIA: juvenile idiopathic arthritis (two samples at different occasions).

**Table 2:** Summary of similar studies in the literature, including the present study results, with aqueous and/or vitreous humor analysis in patients with uveitis.

Description	Number of individuals	Uveitis Classification	PCR Positivity	Investigated Pathogens
Rothova et al. (14)	152 patients 40 controls	Posterior	Overall positivity:* 29% (44/152) 34% (15/44 PCR; AH)	*T. gondii* HSV, VZV, CMV
Santos et al. (12)	27 patients	Anterior Intermediate Posterior Panuveitis	19.2% (5/26; AH) 30% (6/20; VH)	*T. gondii* HSV, VZV, CMV *M. tuberculosis*
Kongyai et al. (21)	80 patients	Posterior Panuveitis	30% (24/80) 35% (19/54; AH) 19% (5/26; VH)	*T. gondii* HSV, VZV, CMV
Scheepers et al. (27)	159 patients	Anterior Intermediate Posterior Panuveitis	59% * (94/159; AH and/or VH)	*T. gondii* HSV, VZV, CMV *M. tuberculosis*
Errera et al. (30)	42 patients 16 controls	Anterior	Overall positivity:* 38% (16/42; AH) 35.7% (5/14 PCR; AH)	HSV, VZV, CMV
Santos et al. (31)	55 patients 12 controls	Posterior (toxoplasmic retinochoroiditis)	37.2% (16/43; AH)	*T. gondii*
Chronopoulos et al. (32)	45 patients	Anterior Intermediate Posterior Panuveitis	48.9% (22/45; AH)	*T. gondii* HSV, VZV,CMV, EBV
Kumar et al. (33)	126 patients 100 controls	Anterior Intermediate Posterior Panuveitis	32.4% (12/37; AH) 23.6% (21/89; VH)	*T. gondii* HSV,VZV, CMV
Calvo et al. (34)	14 patients	Posterior (Acute retinal necrosis)	78.5% (11/14; AH)	HSV, VZV
Elyashiv et al. (35)	28 patients	Posterior	57% (16/28; AH)	*T. gondii* HSV, VZV, CMV
Present study	28 patients	Anterior Posterior Panuveitis	41.7% (5/12; AH) 31.2% (5/12; VH)	*T. gondii* HSV, CMV, EBV

PCR = polymerase chain reaction; AH = aqueous humor; VH = vitreous humor; *T. gondii* = *Toxoplasma gondii*; HSV = herpes *simplex* virus; VZV = *Varicela zoster* virus; CMV = cytomegalovirus; EBV = *Epstein-Barr* virus; *M. tuberculosis* = *Mycobacterium tuberculosis.*

* These studies included other methods beyond PCR analysis.
